# Hydrogen-Sulfide-Mediated PpAOS3-JA Module Provides Insight into Salt Stress Resistance in Peach

**DOI:** 10.3390/plants14101477

**Published:** 2025-05-15

**Authors:** Xiaolan Gao, Miao Li, Qingtao Gong, Guixiang Li, Haixiang Yu, Xiaomin Dong, Xiaoyou Wang, Zheng Gong, Zhongtang Wang, Yuansong Xiao, Anning Zhang

**Affiliations:** 1 Shandong Institute of Pomology, Shandong Academy of Agricultural Sciences/State Key Laboratory of Efficient Utilisation of Nutrient Resources, Tai’an 271018, China; gxlsdsgss@163.com (X.G.); limiao6543@163.com (M.L.); gongzheng.1984@163.com (Q.G.); liguixiang-2010@163.com (G.L.); dxm1209@163.com (X.D.); sdgss213@163.com (Z.W.); 2 College of Horticulture Science and Engineering, Shandong Agricultural University, Tai’an 271018, China; yhxsdau@163.com (H.Y.); ysxiao@sdau.edu.cn (Y.X.); 3 Mengyin County Fruit Industry Development Service Center, Linyi 276000, China; wxy-76@163.com (X.W.); 13053957568@163.com (Z.G.)

**Keywords:** *Prunus persica*, salt stress, exogenous hydrogen sulfide, antioxidative system, JA

## Abstract

Salt stress is one of the main abiotic stresses that affects peach growth. Hydrogen sulfide has an important role in regulating plant resistance to salt stress. However, the mechanism by which hydrogen sulfide regulates salt stress resistance is currently unclear in peach. Here, we investigated the mechanism by which hydrogen sulfide alleviates salt stress in peach trees. In our study, exogenous hydrogen sulfide enhances the activity of antioxidant enzymes and reduces the accumulation of reactive oxygen species, thereby mitigating salt stress damage to seedlings. Moreover, transcriptome analysis was carried out and an encoding allene oxide synthase gene (AOS), *PpAOS3*, which is highly responsive to hydrogen sulfide, was found. Overexpression of *PpAOS3* increased the root length and jasmonic acid (JA) content and attenuated growth inhibition under salt stress in *Arabidopsis*. NBT and Evans staining showed that *Arabidopsis* overexpressing *PpAOS3* reduces O^2−^ accumulation and cell death under salt stress. Additionally, transcriptome analysis revealed that 10 genes encoding oxidoreductase were upregulated after hydrogen sulfide treatment. RT-qPCR was also performed which showed that these genes were upregulated to different degrees after hydrogen sulfide treatment. In conclusion, a hydrogen-sulfide-mediated *PpAOS3*-JA module significantly contributes to salt resistance in peach. These results can serve as a theoretical basis for utilizing hydrogen sulfide to improve the salt tolerance of peach.

## 1. Introduction

Peach (*Prunus persica* (L.) Batsch) is a deciduous tree or shrub in the family Rosaceae grown all over the world. Peaches are one of the world´s most important fruits [1]. Soil salinity is a major abiotic stress during the growth and development of plants. Salt stress seriously affects plant growth, quality, and yield, with sodium salt being the most common cause of damage [2]. Peach yield productivity tends to decline above a threshold for ECe of 4 dS m^−1^ [3]. Under salt stress, plant height, root length, leaf number, and dry matter quality are reduced, leading to reduced photosynthetic efficiency, accelerated material metabolism, increased aging, and, ultimately, plant death [4]. Some studies have found that the composition of the cytosol and the structure of the cell membrane also change under the influence of salt stress, thus hindering the normal metabolism of plants [5]. In studies on grapevine plants, salt stress was found to reduce the number of branches, branch and root lengths, root volume, and leaf area, as well as the relative water content (RWC) and other morphological and physiological indices, of seedlings [6], and it caused a decrease in leaf area and carbohydrate accumulation [7]. Other studies have proven that when apple trees are stressed by salinity, their leaves turn yellow and wither, leading to premature aging [8]. In addition to plant morphology, cell structure and function have also been found to be affected by salt stress.

Hydrogen sulfide (H_2_S) is an endogenous plant gas signaling molecule that is widely involved in various plant physiological processes and in the regulation of plant responses to stress [9]. Hydrogen sulfide can reduce the damage to peach plants caused by salt water stress by enhancing active oxygen metabolism [10]. When plants were subjected to osmotic stress, hydrogen sulfide was involved in the response to stress by closing stomata and regulating the accumulation of osmotic regulatory substances [11]. Some have shown that hydrogen sulfide treatment increased the enzymatic and non-enzymatic antioxidant capacity of rapeseed oil and kale seedlings, thereby reducing oxidative damage and improving the growth of kale under aluminum stress [12]. Studies have found that NaHS treatment can alleviate oxidative damage in wheat seedlings undergoing high-temperature stress by regulating the antioxidant defense system, thus improving the tolerance of wheat seedlings to high-temperature stress [13]. In addition, hydrogen sulfide treatment enhances the antioxidant capacity of seedlings and alleviates oxidative damage caused by salt stress, thereby improving their tolerance to salt stress to a certain extent [14]. Hydrogen sulfide can reduce oxidative damage caused by salt stress by enhancing the antioxidant enzyme activity of tea tree (*Camellia sinensis*) [15] and can alleviate the decrease in mineral element content and inhibition of plant growth in eggplant (*Solanum melongena*) under salt stress [16]. Furthermore, hydrogen sulfide can improve photosynthesis in *Capsicum annuum* under cold stress by promoting chlorophyll synthesis [17]. Hydrogen sulfide triggers nitric oxide signaling, thereby improving photosynthesis and biomass accumulation in the medicinal plant *Cyclocarya papaliurus* under salt stress [18]. Exogenous hydrogen sulfide treatment can also protect rice seedlings under salt stress by improving the antioxidant capacity of plant tissues and promoting the expression of related protein genes [19].

Plant hormones have been recognized as important endogenous molecules involved in the regulation of plant development and tolerance or sensitivity to various stresses, including salt stress [20]. Allene oxide synthase (AOS) synthesizes precursors of jasmonic acid and plays a key role in the synthesis of jasmonic acid (JA) and biologically active jasmonoyl-isoleucine (JA-Ile) [21]. JA is a key signaling molecule for multiple developmental processes and defense responses in plants. It was found that high salt stress increased bioactive JA in plants, suggesting that higher JA could act as an effective protectant against salt-mediated adverse effects [22]. Similarly, the application of exogenous JA effectively reduced sodium in salt-tolerant rice [23]. In addition, JA treatment restored salt-induced defects in seedling development and photosynthetic activity in crops. Recent studies on the functional roles of phytohormones under unfavorable environmental conditions have finally revealed their potential to confer tolerance under conditions of salt stress [20].

In order to make full use of land resources, planting peach trees in saline–alkali land has broad prospects. Therefore, improving the salt tolerance of peach trees has become an urgent problem to be solved. Hydrogen sulfide is the third gas signal molecule found after NO and CO, and it plays an important role in plant growth and development and alleviating abiotic stress. At present, the effect of exogenous hydrogen sulfide in alleviating abiotic stress has been reported [24]. Hydrogen sulfide has an important role in regulating plant resistance to salt stress. However, the mechanism by which hydrogen sulfide regulates salt stress resistance is currently unclear in peach. We hypothesized that exogenous H₂S enhances salt tolerance through the PpAOS3-mediated JA pathway. Therefore, this study took peach as the experimental material to study the effects of exogenous hydrogen sulfide on the growth, development, and physiological and biochemical characteristics of peach seedlings under salt stress. The exogenous hydrogen sulfide increased the photosynthetic rate of peach seedlings and reduced the accumulation of reactive oxygen species thereby increasing salt resistance. Transcriptome analysis was also carried out and an encoding allene oxide synthase gene, *PpAOS3*, which is highly responsive to hydrogen sulfide, was found. Additionally, overexpression of *PpAOS3* increases root length and JA content and attenuates growth inhibition under salt stress in *Arabidopsis*. NBT and Evans staining showed *Arabidopsis* overexpressing *PpAOS3* reduces O^2−^ accumulation and cell death under salt stress. These research results can serve as a theoretical basis for utilizing hydrogen sulfide to improve the salt tolerance of peach and can provide technical support for peach cultivation in saline–alkaline soil.

## 2. Results

### 2.1. Salt Stress Impairs the Growth of Peach Seedlings

**Figure 1:**
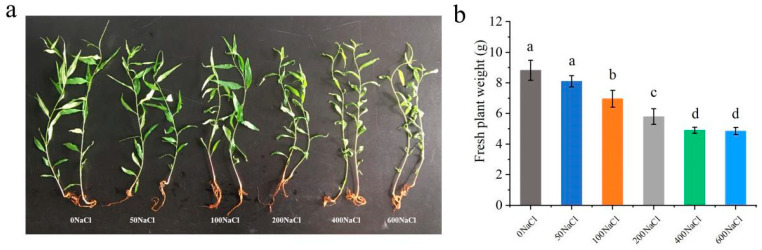
Effects of different concentrations of salt treatment on the growth and fresh weight of peach seedlings: (**a**) growth of peach seedlings; (**b**) fresh weight of peach seedlings. Error bars represent standard deviations of the means (n = 3). Different lowercase letters indicate significant differences among the various treatments (Duncan test, *p* < 0.05).

Increasing NaCl concentrations progressively inhibited peach seedling growth. Salt treatment inhibited root development and plant growth. From 50 to 600 mM NaCl, salt injury intensified in a dose-dependent manner, with the growth of peach plants being inhibited and the fresh weight of the plants gradually decreasing (Figure 1a). Compared with the control, the fresh weight of the 200NaCl (200 mM NaCl) treatment decreased by 34.3% (Figure 1b). We chose 200 mM NaCl, which reduced fresh weight by 34.3% yet caused no mortality in subsequent experiments.

### 2.2. Exogenous Hydrogen Sulfide Alleviates the Decrease in Photosynthetic Rate of Seedlings Under Salt Stress

**Figure 2:**
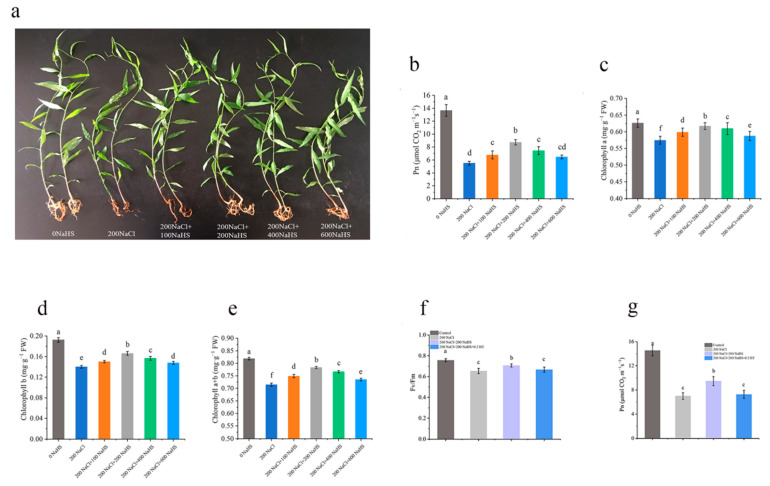
Effects of different concentrations of sodium hydrosulfide treatment on the growth, net photosynthetic rate, and chlorophyll content of peach seedlings under salt stress: (**a**) growth of peach seedlings; (**b**) net photosynthetic rate of peach seedlings; (**c**) chlorophyll a content of peach seedlings; (**d**) chlorophyll b content of peach seedlings; (**e**) chlorophyll a + b content of peach seedlings. Effect of exogenous hydrogen sulfide on photosynthetic characteristics of peach seedlings leaves under salt stress: (**f**) maximum photochemistry efficiency of PSII (Fv/Fm); (**g**) photosynthetic rates (Pn). Error bars represent standard deviations of the means (n = 3). Different lowercase letters indicate significant differences among the various treatments (Duncan test, *p* < 0.05).

Under salt stress of 200NaCl, the net photosynthetic rate and chlorophyll content of the peach seedlings were significantly lower than those of the control. Compared with 200NaCl salt stress, exogenous hydrogen sulfide treatment increased the net photosynthetic rate and chlorophyll content of the leaves under salt stress (Figure 2b). Compared with 200NaCl only, the net photosynthetic rate and chlorophyll a, chlorophyll b, and chlorophyll a + b contents of the peach seedlings significantly increased after the 200NaHS treatment (Figure 2c–e). The Fv/Fm reflects the primary light energy conversion efficiency of the PSII reaction center. Under salt stress, the Fv/Fm and net photosynthetic rate of the peach leaves significantly decreased, while hydrogen sulfide alleviated this reduction (Figure 2f,g). Furthermore, after applying the desulfurizer (HT), the Fv/Fm and net photosynthetic rate decreased again, which shows that exogenous hydrogen sulfide has a crucial role in alleviating damage to the photosynthetic system.

### 2.3. Exogenous Hydrogen Sulfide Alleviates Salt Stress Damage in Seedlings by Reducing ROS Production

**Figure 3:**
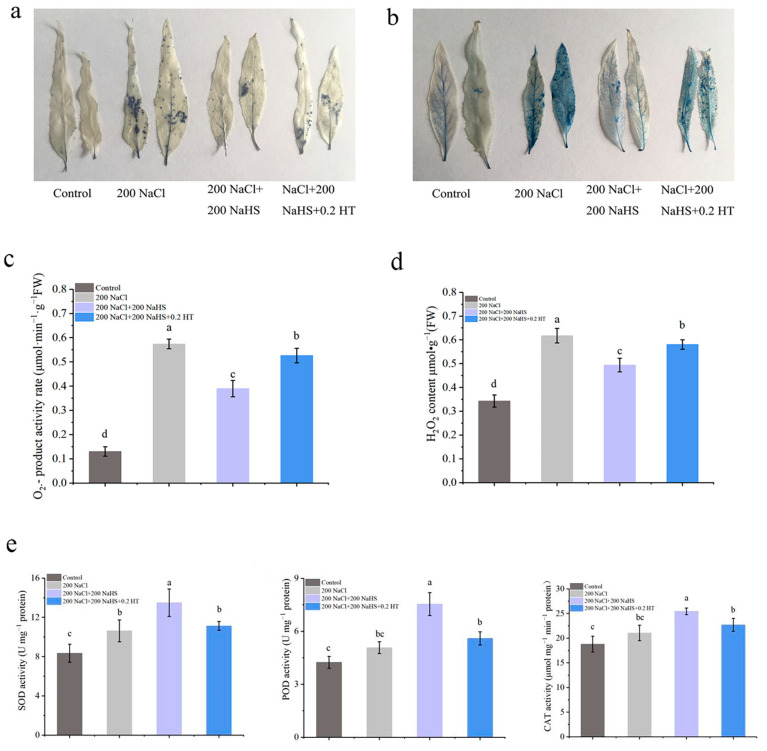
(**a**) Distribution of superoxide anion radicals (O^2−^) visualized by nitroblue tetrazolium (NBT); (**b**) staining with Evans blue to show areas of cell death; (**c**) superoxide radical ion (O^2−^) product activity rate; (**d**) hydrogen peroxide (H_2_O_2_) content; (**e**) superoxide dismutase (SOD), peroxidase (POD), and catalase (CAT) activities. Error bars represent standard deviations of the means (n = 3). Different lowercase letters indicate significant differences among the various treatments (Duncan test, *p* < 0.05).

Salt stress can cause oxidative damage and cell injury. Under salt stress, exogenous hydrogen sulfide reduced the damage caused by reactive oxygen species production in peach leaves, and the number of dead cells was significantly reduced (Figure 3a,b). In addition, when a hydrogen sulfide scrubber (HT) is added under salt stress, the oxidative damage of seedlings is aggravated, indicating that hydrogen sulfide plays a certain role in alleviating oxidative damage under salt stress (Figure 3a,b). We measured the superoxide anion formation rate and hydrogen peroxide content of peach roots after the application of exogenous hydrogen sulfide under salt stress. The superoxide anion production rate and hydrogen peroxide content in peach roots significantly increased under salt stress, but the application of exogenous hydrogen sulfide significantly decreased the superoxide anion production rate and hydrogen peroxide content in peach roots. Again, the use of hydrogen sulfide scavengers can partially eliminate this effect (Figure 3c,d).

The antioxidant enzyme system in plants plays an important role in clearing ROS. To this end, we examined the protective enzyme activity in peach root lines under the various treatment conditions. The results show that the antioxidant enzyme activity of the peach roots increased to some extent under the salt stress compared with the control conditions, which may indicate a stress response. Under salt stress conditions, exogenous application of hydrogen sulfide further improved the antioxidant oxidase activity of peach roots to enhance root resistance to salt stress (Figure 3e). These results suggest that exogenous hydrogen sulfide could reduce the damage caused by salt stress in peach trees (Figure 3d,e).

### 2.4. Transcriptome Analysis of Peach Seedlings Treated with Hydrogen Sulfide Under Salt Stress and Salt Stress Only

**Figure 4:**
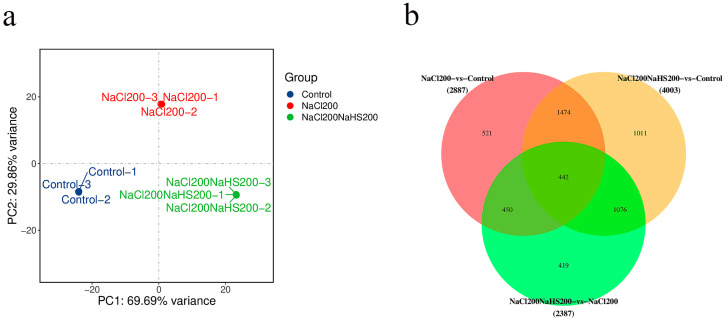
(**a**) Principal component analysis of the three transcriptomes; (**b**) Venn diagram analysis of all induced unigenes.

To further assess the role of hydrogen sulfide in alleviating salt stress, a transcriptome was performed on the treatment samples. We constructed three cDNA libraries after treating peach seedling with 200 mM NaCl (200NaCl) and 200 mM NaCl with 200 mM NaHS (200NaCl200NaHS) for 24 h using water as a control. This analysis completed the sequencing of nine samples of the reference transcriptome and obtained a total of 58.76 G of CleanData; the effective data volume of each sample was distributed in the range of 5.67–6.97 G, the distribution of the Q30 bases was in the range of 90.15–92.45%, and the average GC content was 45.91%. The clean reads were obtained with Q30 > 92.45% (control), 92.27% (200NaCl), and 92.42% (200NaCl200NaHS) by removing the adaptor and low-quality sequences (Table S1). To determine the accuracy and confidence of the data, we performed principal component analysis (PCA). The PCA showed good repeatability on the three biological replicates, and there was a great difference between the 200NaCl and 200NaCl200NaHS (Figure 4a). In addition, we obtained the differentially expressed genes (DEGs) among the three treatments. As expected, each set of comparisons had a higher number of differentially expressed genes, indicating that the different treatments induced the gene expression changes at the transcript level (Figure 4b).

### 2.5. Differentially Expressed Genes Between Seedlings Treated with Hydrogen Sulfide Under Salt Stress and Salt Stress Only

**Figure 5:**
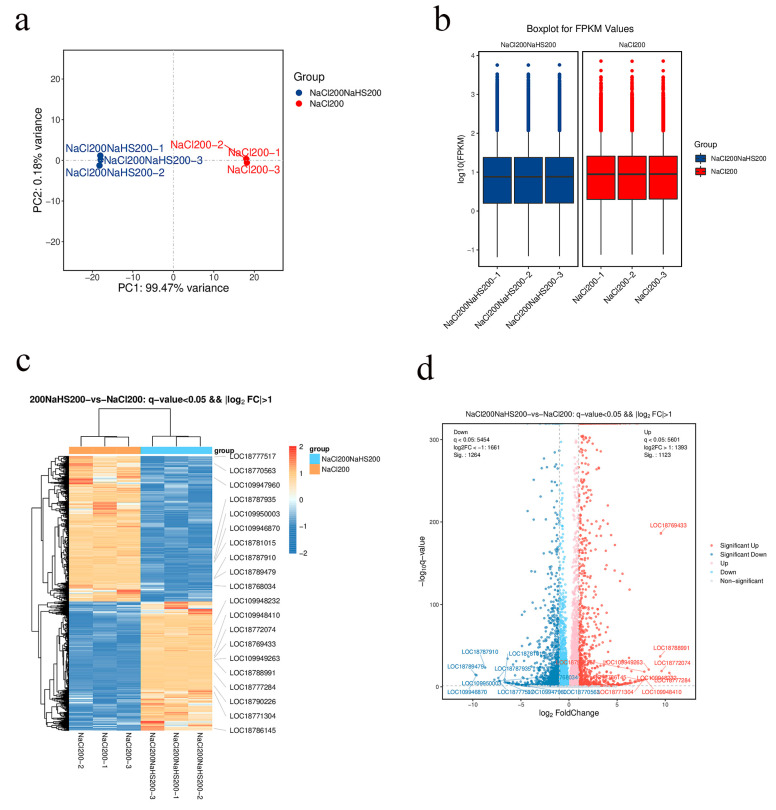
(**a**) Principal component analysis of two transcriptomes (200NaCl vs. 200NaCl200NaHS); (**b**) NaCl200NaHS200 vs. NaCl200 FPKM boxplot; (**c**) hierarchical cluster map of the DEGs based on the FPKM values, with three biological replicates of each treatment included in the analysis; (**d**) volcano map of the DEGs from the comparison of the 200NaCl and 200NaCl200NaHS treatments.

Our results suggest that exogenous hydrogen sulfide has an important role in alleviating salt stress. Given these results, we focused on transcriptome profile differences in comparison of the 200NaCl and 200NaCl200NaHS treatments. In order to study the effects of the various treatments on the gene transcription profiles of the peach seedlings under salt stress, principal component analysis (PCA) was performed on the transcriptome data. The FPKM values were calculated, and the Pearson correlation coefficients (PCCs) of all of the expressed genes in all of the samples were calculated based on the FPKM values (Figure 5a,b). The results show that replicates of the same treatment were close to each other, but there were significant differences in the transcription levels of the various treatment samples, indicating that exogenous hydrogen sulfide significantly changed the gene transcription profile of the peach seedlings under salt stress. Furthermore, the log2 (fold-change) value was used for the hierarchical clustering of the DEGs, and there were 2387 genes in response to the hydrogen sulfide and salt stress, of which 1123 were upregulated and 1264 were downregulated (Table S2). Whole expression profiling of the DEGs was also performed in order to identify the most significant 20 DEGs (Figure 5c,d).

### 2.6. PpAOS3 Is Highly Responsive to Exogenous Hydrogen Sulfide Under Salt Stress

**Figure 6:**
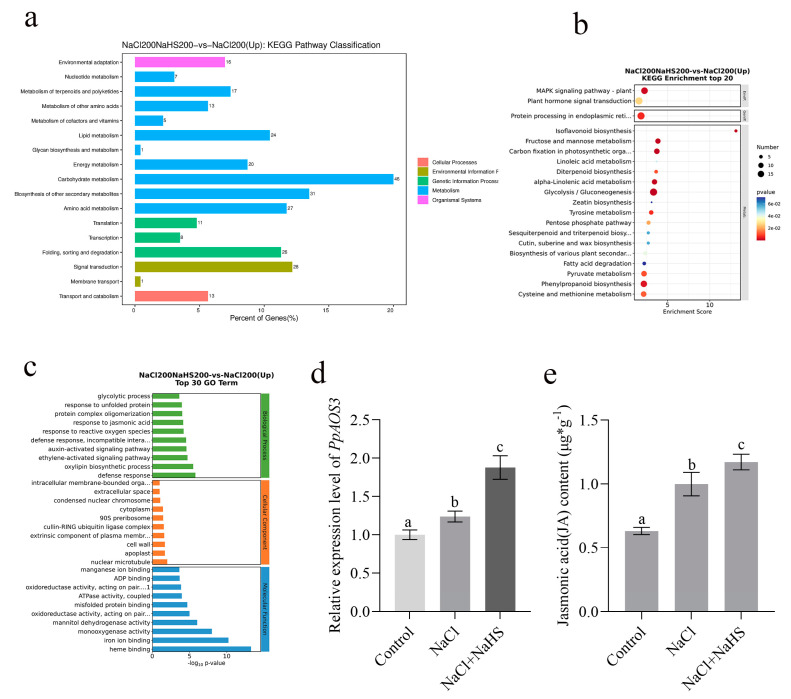
GO and KEGG enrichment analyses of DEGs: (**a**) KEGG pathway classification of the 200NaCl vs. 200NaCl200NaHS comparison; (**b**) top 20 KEGG entries that were significantly enriched in DEGs identified from the 200NaCl vs. 200NaCl200NaHS comparison; (**c**) top 30 GO entries that were significantly enriched in DEGs identified from the 200NaCl vs. 200NaCl200NaHS comparison; (**d**) relative expression of *PpAOS3* under the various treatments; (**e**) jasmonic acid content of peach seedlings under the various treatments. Error bars represent standard deviations of the means (n = 3). Different lowercase letters indicate significant differences among the various treatments (Duncan test, *p* < 0.05).

We conducted a KEGG enrichment analysis for the main pathways involved in DEGs, and the results show that 81 pathways were upregulated by hydrogen sulfide (Table S3). These pathways involve processes such as carbohydrate metabolism and amino acid metabolism (Figure 6a). The top 20 pathways obtained from the KEGG enrichment analysis show that the biosynthesis of various plant secondary metabolites—including phenylpropanoid biosynthesis, plant–pathogen interaction, isoflavonoid biosynthesis, tyrosine metabolism, alpha-linolenic acid metabolism, and the MAPK signaling pathway—are important pathways that underwent significant changes (Figure 6b).

To understand the biological pathways of the DEGs under the various treatments, we performed a Gene Ontology (GO) enrichment analysis and explained their functions to better characterize the DEGs. We annotated 1529 and 1464 GO terms, filtered and classified them, and divided them into three levels according to the functional classification. The first level contains the following three terms: biological processes, cell components, and molecular functions. Level 2 contains 64 terms, including bioadhesion and cell and binding. The third level contains tens of thousands of conventional enrichment provisions (Table S4). According to the first 30 GO enrichment items, it was found that the DEGs under hydrogen sulfide and salt stress were involved in cell wall biogenesis, response to reactive oxygen species, cell walls, redox enzyme activity, and action on paired donors (Figure 6c).

Given the potential role of redox pathways from the enrichment analysis, we focused on genes encoding redox functions that were significantly upregulated in hydrogen sulfide treatment. In total, 1123 DEGs that were upregulated were selected, including an allene oxide synthase 3 (PpAOS3) (Table S2). *PpAOS3* is highly responsive to hydrogen sulfide and is a key gene in the jasmonate acid (JA) synthesis pathway. Previous studies suggest that the application of JA could provide tolerance against biotic and abiotic stresses, such as salinity, temperature stress, and heavy metal stress [25,26]. Consequently, we also performed a RT-qPCR assay, and, again, its expression was significantly upregulated after hydrogen sulfide (Figure 6d). Furthermore, the jasmonic acid content of the hydrogen-sulfide-treated peach seedlings under salt stress was higher than that in the control (Figure 6e), which also demonstrates the role of *PpAOS3* in the resistance to salt stress.

### 2.7. Phenotypic Characterization of OE-PpAOS3 Arabidopsis

**Figure 7:**
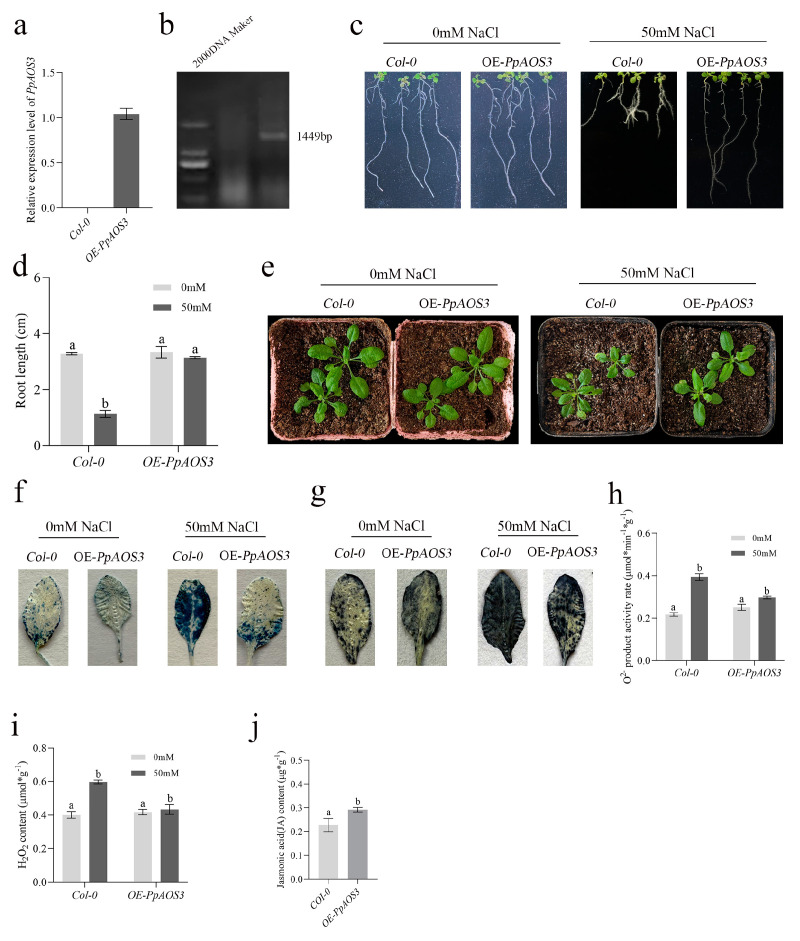
(**a**,**b**) Identification of *PpAOS3*-overexpressing *Arabidopsis*; (**c**) root architecture of *Arabidopsis* seedlings of different genotypes treated with various concentrations of NaCl; (**d**) root length of *Arabidopsis* seedlings of different genotypes treated with various concentrations of NaCl; (**e**) phenotypes of *Arabidopsis* seedlings treated with various concentrations of NaCl in artificial vegetative soil (scale bar: 2 cm); (**f**) Evens staining of *Arabidopsis* leaves treated with various concentrations of salt; (**g**) NBT staining of *Arabidopsis* leaves treated with various concentrations of salt; (**h**,**i**) determination of the rate of O^2−^ production and H_2_O_2_ content in *Arabidopsis;* (**j**) determination of the JA content in *Arabidopsis.* Different lowercase letters indicate significant differences among the various treatments.

To confirm that the salt stress resistance was induced by the transcriptional activation of *PpAOS3*, the functions of *PpAOS3* were investigated. Heterologous gene expression can reflect the role of a gene in native plants to a certain extent. Thus, we constructed overexpressing Arabidopsis lines and verified the expression of *PpAOS3* by PCR and RT-qPCR (Figure 7a,b). The OE-PpAOS3 lines and *COl-0* Arabidopsis were treated with various concentrations of NaCl in MS medium. It could be seen that there was no significant difference in root length between *COl-0* and the overexpression lines with the 0 mM NaCl treatment, whereas roots were inhibited in *COl-0* at 50 mM NaCl (Figure 7c,d). Furthermore, the slow growth in *COl-0* was present under salt stress in potting experiments (Figure 7e). Accumulation of ROS is a characteristic of salt stress damage in plants [27,28]. O^2−^ accumulation and cell death in *Arabidopsis*, examined by NBT and Evans staining, showed results similar to their phenotypes (Figure 7f,g). H_2_O_2_ and O^2−^ production were detected, further supporting the function of *PpAOS3* in salt stress resistance (Figure 7h,i). Given the function of *PpAOS3*, we also examined the JA content in Arabidopsis under salt stress, and, as expected, the JA content of the overexpression lines was significantly higher than in *COl-0* (Figure 7j). Collectively, these results suggest that *PpAOS3* enhances the JA synthesis pathway and thereby increases salt stress resistance in plants.

### 2.8. Encoding Oxidoreductase Genes’ Response to Hydrogen Sulfide and Its Expression Patterns

**Figure 8:**
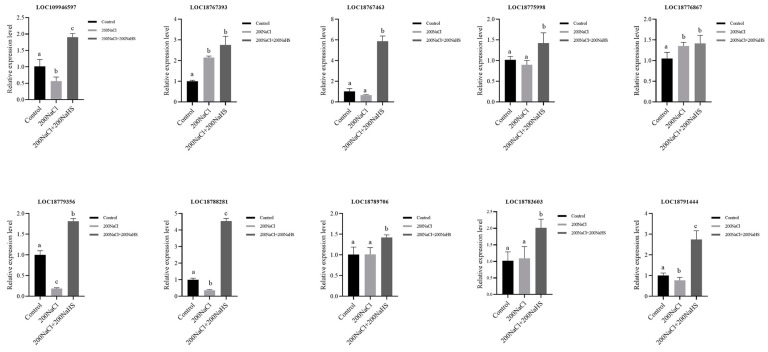
Relative expression of the encoding oxidoreductase genes that were upregulated with the 200NaCl200NaHS treatment. Different lowercase letters indicate significant differences among the various treatments.

Reactive oxygen species (ROS) are highly reactive molecules that can cause oxidative damage to proteins [29]. It has long been known that, under salt stress conditions, the levels of ROS in plant tissues can dramatically elevate [30]. Previous studies have shown that in plants, some oxidoreductase proteins can be involved in the scavenging of ROS. In order to understand the expression of the encoding oxidoreductase genes that responded to hydrogen sulfide under salt stress, we carried out the GO enrichment analysis again and selected eight oxidoreductases genes (*LOC109946597*; *LOC18767393*; *LOC18767463*; *LOC18775998*; *LOC18776867*; *LOC18779356*; *LOC18788281*; *LOC18789706*; *LOC18790620*; *LOC18791444*) that were upregulated in response to hydrogen sulfide (Table S5). Subsequently, in order to clarify the expression patterns of these genes in the process of salt stress resistance, their expression levels were detected by RT-qPCR, and the results show that all genes were upregulated to varying degrees after the hydrogen sulfide treatment (Figure 8). These results are helpful for further elucidating the mechanism by which oxidoreductase alleviates salt stress damage.

## 3. Discussion

More than 6% of the world’s land area is affected by soil salinization [31]. Soil salinity is increasing because of irrigation, improper application of fertilizers, and industrial pollution. High salinity is usually due to high concentrations of Na^+^, causing hypertonic and highly ionized conditions that prevent plants from absorbing water and nutrients from the soil [32,33]. Salt stress can lead to ionic, osmotic, and secondary stresses in plants, especially oxidative stress. Over the past decades, the effects of salt stress on plants and the signaling pathways by which plants mitigate salt stress have been studied by physiological and biochemical means [34].

Photosynthesis is the main way plants synthesize nutrients [35]. Salt stress can damage the reaction center of photosynthetic system II, thus inhibiting photosynthetic electron transfer efficiency and photosynthetic activity [36,37]. We found that plant growth was significantly inhibited under salt stress, and the plant fresh weight, leaf chlorophyll content, and net photosynthetic rate decreased (Figure 1). However, exogenous application of hydrogen sulfide effectively alleviated the salt stress damage to peach seedlings. Changes in chlorophyll content directly affect the capture and conversion of light energy by chloroplasts, since chlorophyll is a component of the protein complex involved in light energy absorption and conversion [38]. Chlorophyll fluorescence parameters reflect the absorption, transmission, dissipation, and distribution of light energy in the plant photosynthetic system [39], and the Fv/Fm reflects the primary light energy conversion efficiency of the PSII reaction center [40]. Under salt stress, the exogenous hydrogen sulfide treatment alleviated the damage to the peach seedling’s photosynthetic mechanism to a certain extent and reduced the decline in the primary light energy conversion efficiency of the PSII reaction center (Figure 2f,g).

Reactive oxygen species (ROS) are secondary metabolites of plant respiration and mainly composed of oxidizing substances such as superoxide anion (O^2−^) and hydrogen peroxide (H_2_O_2_). Under normal conditions, there is a dynamic equilibrium state between the generation and removal of ROS [41,42]. Salt stress can increase contents of superoxide anion, hydrogen peroxide, and other superoxide free radicals; destroy the dynamic balance of the oxidation–reduction; and cause damage to plants. It was found that under salt stress conditions, the O^2−^ content in grape leaves increased with the increase in the number of stress days, resulting in the gradual aggravation of the membrane lipid peroxidation [43]. Another study revealed that the H_2_O_2_ content of Calendula significantly increased after salt stress [44]. Excessive reactive oxygen species can interfere with the normal metabolism of intracellular biomolecules, polysaccharides, lipids, proteins, and nucleic acids, accelerating cellular senescence and, in severe cases, leading to plant death [45]. Our study reveals that under salt stress, after the application of exogenous hydrogen sulfide, the antioxidant oxidase activity of peach seedling roots increased (Figure 3c,d), the active oxygen scavenging ability significantly increased, the content of active oxygen significantly decreased, and the degree of damage to leaf cells significantly decreased (Figure 3a,b,e). To enable plants to cope with the salt-stress-induced ROS accumulation, the antioxidant oxidase system in the plants together with the antioxidant nonenzymatic substances form a complete antioxidant system that plays an important role in ROS scavenging. Common antioxidant enzymes include superoxide dismutase (SOD), peroxidase (POD), and catalase (CAT), which are distributed in various parts of plants and have different reactive oxygen scavenging abilities [46]. In this study, it was found that when peach seedlings were subjected to salt stress, the clearance of the ROS accelerated, and the balance of the ROS metabolism was maintained by adjusting the activity of the above enzymes.

The principal component analysis by high-throughput sequencing suggests that peach seedlings are sensitive to hydrogen sulfide under salt stress. We calculated the Pearson correlation coefficients (PCCs) for all expressed genes in the samples based on the FPKM values, which showed that there were significant differences in the transcription levels of the various treatment samples. Additionally, 2387 DEGs responsive to hydrogen sulfide were identified, of which 1123 were upregulated and 1264 were downregulated. The KEGG and GO analyses showed that the biosynthesis of various plant secondary metabolites—including phenylpropanoid biosynthesis, plant–pathogen interaction, isoflavonoid biosynthesis, tyrosine metabolism, alpha-linolenic acid metabolism, and MAPK and ROS pathways—were upregulated by hydrogen sulfide. These results provide transcript-level insights for a preliminary understanding of the molecular mechanisms by which hydrogen sulfide mitigates salt stress.

Allene oxide synthase (AOS) is a class of genes involved in the JA pathway in plants and is a key synthase for JA precursors. Previous studies have shown that the JA content increases in biotic and abiotic plant stresses, as well as the expression level of AOS [47]. In this study, peach allene oxide synthase 3 (*PpAOS3*) is highly responsive to hydrogen sulfide. Overexpression of *PpAOS3* increased the root length and JA content and attenuated growth inhibition under salt stress in *Arabidopsis*. NBT and Evans staining showed that *Arabidopsis* overexpressing *PpAOS3* reduces O^2−^ accumulation and cell death under salt stress (Figure 7). Our research suggests that *PpAOS3* has an important role in alleviating salt stress and provides a theoretical basis for further studies on the molecular mechanism of hydrogen-sulfide-mediated salt resistance in the future. In addition, the increase in JA content may have a negative effect on plant growth. Therefore, how plants weigh the balance between resistance and growth still needs to be investigated.

In a study of wheat, it was found that exogenous hydrogen sulfide can significantly increase the activity of oxidoreductase, reduce the accumulation of reactive oxygen species, maintain the stability of cell membranes, and effectively improve the antioxidant capacity of seedlings under drought conditions [48]. Here, the transcriptome analysis showed that 10 encoding oxidoreductase genes were significantly upregulated after hydrogen sulfide; this represents the possibility that hydrogen sulfide may reduce the damage caused by salt stress by enhancing the antioxidant enzyme system in plants. However, the function of most oxidoreductases in salt resistance remains unclear, and their molecular mechanisms need to be further investigated.

## 4. Materials and Methods

**Test Material**

The experiment was carried out at the Shandong Fruit Research Institute. Seeds of the “Mountain Peach” cultivar, which had been stored in a sand deposit for 3 months, were sown in a seedling tray. After normal management for a period of time, plants at the 8-leaf stage with strong and consistent growth were selected for transplantation. Each test pot was a plastic pot (inner diameter of 17 cm and height of 15 cm), and 5 kg of the test soil (loam:vermiculite:turf:organic fertilizer = 1.5:1:1:0.5) was mixed and packed to be 3–4 cm away from the rim of the pot. One plant was planted in each pot, the soil was moistened after filling the pot, and normal management was carried out.

**Treatments**

Screening the Optimal Sodium Chloride Treatment Concentration for Salt Stress. To achieve the optimal effect of salt stress, natural peach salt stress conditions were simulated. First, the concentration of the sodium chloride treatment was screened. The NaCl concentrations of the treatment groups were as follows: 0 mM (μmol/L, micromole per L) NaCl (water control), 50NaCl, 100NaCl, 200NaCl, 400NaCl, and 600NaCl. The peach plants were randomly arranged, with 15 plants per treatment, and were maintained with normal management. After 5 days of treatment, the phenotypes of the plants were observed and photographed. The fresh weight of each treated plant was measured with an electronic balance, the appropriate concentration of sodium chloride was determined for continued tests, and the measurements were repeated 3 times per treatment.

**Screening and Determining the Optimal Concentration of Exogenous Hydrogen Sulfide under Salt Stress**

The optimal concentration of hydrogen sulfide was screened by adding sodium hydride (a hydrogen sulfide donor) under 200NaCl salt stress. The concentrations of the sodium hydrosulfide treatment groups were as follows: 0NaHS (0 mM NaHS) + 0NaCl (water control), 100NaHS + 200NaCl, 200NaHS + 200NaCl, 400NaHS + 200NaCl, and 600NaHS + 200NaCl. The plants were randomly arranged, with 15 plants per treatment, and they were maintained with normal management. After 5 days of treatment, the net photosynthetic rate and chlorophyll content of the plants in each treatment group were assessed to determine the appropriate application concentration of exogenous hydrogen sulfide, and the measurements were repeated 3 times per treatment.

**Alleviating Effect of Exogenous Hydrogen Sulfide on Peach Seedlings under Salt Stress**

The treatment groups’ concentrations were as follows: water control, 200NaCl treatment, 200NaCl + 200NaHS treatment, and hydrogen sulfide remover (THiotaurine HT) treatment 200NaCl + 200NaHS + 0.2 mMHT. The plants were randomly arranged, with 15 plants per treatment, and they were maintained with normal management. The oxidative damage, cell death, superoxide anion production rate, hydrogen peroxide content, root protective enzyme activity, leaf net photosynthetic rate, fluorescence parameters, and root configuration parameters were determined. The measurements were repeated 3 times per treatment.

**Determination of Chlorophyll Content in Leaves.**

Chlorophyll and carotenoids were extracted with 95% ethanol, and the absorbances of chlorophyll a and chlorophyll b at 663.3 nm and 646.8 nm were determined by spectrophotometry [49].

**Determination of the Photosynthetic Rate and Fluorescence Parameters of Peach Seedling Leaves**

CIRAS-3 portable photosynthesis systems (PPSystens, Stotfold, UK) were used to measure leaf gas exchange parameters from 9:00 a.m. to 11:00 a.m. The leaf chlorophyll content and net photosynthetic rate (Pn) were measured in the middle of each treated plant. The maximum photochemistry efficiency of PSII (Fv/Fm) was determined by a continuous excitation fluorimeter (HandyPEA, Hansatech, Pentney, UK).

**Determination of the Root System Configuration Parameters**

The complete root system of each plant was removed, and after being brought back to the laboratory, the roots were rinsed, and the root configuration parameters were determined by the professional WinRHIZO (V2.01, scanner resolution of 600 dpi) root analysis system. The root configuration parameters included the total surface area (cm^2^), average root diameter (cm), and total root length (m).

**Determination of Reactive Oxygen Species Levels**

We determined the O^2−^ production rate based on the methods described in Elstner and Heupel [50], as well as the H_2_O_2_ content based on the methods described in Patterson et al. [51], with appropriate adjustments. Histochemical Staining Method for O^2−^ and H_2_O_2_.

The nitroblue tetrazolium (NBT) staining was based on the methods described in Hu et al. [52], with appropriate adjustments.

**Determination of the Roots Protective Enzyme Activity**

The superoxide dismutase (SOD) activity was determined based on the methods described in Chen Yizhu et al. [53]. The peroxidase (POD) activity was determined based on the methods described in Omran [54]. The catalase (CAT) activity was determined based on the methods described in Kar et al. [55].

**Determination of Leaf Cell Death**

The leaves of the peach seedlings were stained by the Evans blue staining method and soaked in 0.25% (*w/v*) Evans blue solution for 24 h. The leaves were removed, cleaned with pure water, excess water absorbed, and boiled in a solution of anhydrous ethanol:glycerol (4:1) until the base color of the leaves was white [56]. Photographs were taken to record the staining results.

***Arabidopsis* transformation and Characterization of transgenic *Arabidopsis***

The *PpAOS3* coding sequence was cloned using the primers listed in the Table S6 and fused with the pCambia1300 vector. The recombinant vectors were introduced into Agrobacterium solution (OD:0.6) with 50 ug/mL kanamycin. The floral dipping method was used to create transgenic plants. Phenotyping was performed using T4 generation plants.

**JA quantification**

The JA content in each sample was analyzed using an HPLC-MS/MS system (Waters, Milford, MA, USA) (HPLC: Shim-pack UFLC SHIMADZU CBM30A system; MS: Applied Biosystems 4500 Triple Quadrupole (Waters, Milford, MA, USA)) [57].

**RNA-seq analysis**

For the RNA-seq, the total RNA was extracted from the root using an RNAprep Pure Plant Kit (Tiangen, Beijing, China), with three biological replicates for each tissue. This library was sequenced using the Illumina HiSeq X Ten platform to generate 150 bp paired-end reads. HISAT2 (version 2.0.4) was used to locate clean reads of the peach genome (GCF_000346465.2, NCBI). The FPKM value of each gene was calculated using Cufinks (v2.1). DESeq R software (v2.01) was used for the differential expression analysis. The criterion for a significant difference in expression was *p* < 0.05. In order to visualize gene expression patterns in the different populations and samples, the differentially expressed genes (DEGs) for hierarchical clustering analysis. Based on the hypergeometric distribution, R was used for the GO enrichment and KEGG enrichment analyses of the DEGs [58,59].

**RNA extraction and gene expression analysis**

The total RNA was extracted with an RNA extraction kit (Tiangen, Beijing, China), and the first cDNA was synthesized with a synthesis kit (Takara, Dalian, China). RT-qPCR was performed on the ABI7500 system using SYBR premix ExTaq (Takara, Dalian, China), with the following procedure: 95 °C for 5 min, followed by 40 cycles at 95 °C for 15 s, 60 °C for 10 s, and 72 °C for 20 s. Using the primers listed in Table S6. The relative expression level was calculated by the 2^−ΔΔCT^ method [60].

**Data Processing and Analysis**

Origin version 9.8 was used to conduct all statistical analyses. Duncan multiple range tests, which are included in SPSS version 20.0, were performed to detect statistically significant differences in the mean values (IBM SPSS, Chicago, IL, USA). The threshold for statistical significance used for all tests was *p* < 0.05.

## Data Availability

All the data and methods in this study are available from the corresponding author and the first author. All transcriptome data have been uploaded to the NCBI website (https://www.ncbi.nlm.nih.gov/. URL: accessed on 26 February 2024) under BioProject PRJNA1080967, and the transcriptome data already openly available.

## Supplementary Materials

The following supporting information can be downloaded at: https://www.mdpi.com/article/10.3390/plants14101477/s1, Table S1: The clean data summary of transcriptome; Table S2: The different expressed genes of 200NaCl200NaHS-vs-200NaCl comparison; Table S3: The up-regulated pathway in KEGG enrichment of 200NaCl200NaHS-vs-200NaCl comparison; Table S4: The GO enrichment of 200NaCl200NaHS-vs-200NaCl comparison; Table S5: The up-regulated oxidoreductase genes in GO enrichment; Table S6: The priemers used in this study.
